# Development and Assessment of a Clinical Calculator for Estimating the Likelihood of Recurrence and Survival Among Patients With Locally Advanced Rectal Cancer Treated With Chemotherapy, Radiotherapy, and Surgery

**DOI:** 10.1001/jamanetworkopen.2021.33457

**Published:** 2021-11-08

**Authors:** Martin R. Weiser, Joanne F. Chou, Ajaratu Keshinro, William C. Chapman, Philip S. Bauer, Matthew G. Mutch, Parag J. Parikh, Andrea Cercek, Leonard B. Saltz, Marc J. Gollub, Paul B. Romesser, Christopher H. Crane, Jinru Shia, Arnold J. Markowitz, Julio Garcia-Aguilar, Mithat Gönen

**Affiliations:** 1 Colorectal Service, Department of Surgery, Memorial Sloan Kettering Cancer Center, New York, New York; 2 Department of Epidemiology and Biostatistics, Memorial Sloan Kettering Cancer Center, New York, New York; 3 Department of Surgery, Washington University in St Louis, St Louis, Missouri; 4 Department of Radiation Oncology, Washington University in St Louis, St Louis, Missouri; 5 Department of Medicine, Memorial Sloan Kettering Cancer Center, New York, New York; 6 Department of Radiology, Memorial Sloan Kettering Cancer Center, New York, New York; 7 Department of Radiation Oncology, Memorial Sloan Kettering Cancer Center, New York, New York; 8 Department of Pathology, Memorial Sloan Kettering Cancer Center, New York, New York, New York

## Abstract

**Importance:**

Predicting outcomes in patients receiving neoadjuvant therapy for rectal cancer is challenging because of tumor downstaging. Validated clinical calculators that can estimate recurrence-free survival (RFS) and overall survival (OS) among patients with rectal cancer who have received multimodal therapy are needed.

**Objective:**

To develop and validate clinical calculators providing estimates of rectal cancer recurrence and survival that are better for individualized decision-making than the American Joint Committee on Cancer (AJCC) staging system or the neoadjuvant rectal (NAR) score.

**Design, Setting, and Participants:**

This prognostic study developed risk models, graphically represented as nomograms, for patients with incomplete pathological response using Cox proportional hazards and multivariable regression analyses with restricted cubic splines. Because patients with complete pathological response to neoadjuvant therapy had uniformly favorable outcomes, their predictions were obtained separately. The study included 1400 patients with stage II or III rectal cancer who received treatment with chemotherapy, radiotherapy, and surgery at 2 comprehensive cancer centers (Memorial Sloan Kettering [MSK] Cancer Center and Siteman Cancer Center [SCC]) between January 1, 1998, and December 31, 2017. Patients from the MSK cohort received chemoradiation, surgery, and adjuvant chemotherapy from January 1, 1998, to December 31, 2014; these patients were randomly assigned to either a model training group or an internal validation group. Models were externally validated using data from the SCC cohort, who received either chemoradiation, surgery, and adjuvant chemotherapy (chemoradiotherapy group) or short-course radiotherapy, consolidation chemotherapy, and surgery (total neoadjuvant therapy with short-course radiotherapy group) from January 1, 2009, to December 31, 2017. Data were analyzed from March 1, 2020, to January 10, 2021.

**Exposures:**

Chemotherapy, radiotherapy, chemoradiotherapy, and surgery.

**Main Outcomes and Measures:**

Recurrence-free survival and OS were the outcome measures, and the discriminatory performance of the clinical calculators was measured with concordance index and calibration plots. The ability of the clinical calculators to predict RFS and OS was compared with that of the AJCC staging system and the NAR score. The models for RFS and OS among patients with incomplete pathological response included postoperative pathological tumor category, number of positive lymph nodes, tumor distance from anal verge, and large- and small-vessel venous and perineural invasion; age was included in the risk model for OS. The final clinical calculators provided RFS and OS estimates derived from Kaplan-Meier curves for patients with complete pathological response and from risk models for patients with incomplete pathological response.

**Results:**

Among 1400 total patients with locally advanced rectal cancer, the median age was 57.8 years (range, 18.0-91.9 years), and 863 patients (61.6%) were male, with tumors at a median distance of 6.7 cm (range, 0-15.0 cm) from the anal verge. The MSK cohort comprised 1069 patients; of those, 710 were assigned to the model training group and 359 were assigned to the internal validation group. The SCC cohort comprised 331 patients; of those, 200 were assigned to the chemoradiotherapy group and 131 were assigned to the total neoadjuvant therapy with short-course radiotherapy group. The concordance indices in the MSK validation data set were 0.70 (95% CI, 0.65-0.76) for RFS and 0.73 (95% CI, 0.65-0.80) for OS. In the external SCC data set, the concordance indices in the chemoradiotherapy group were 0.71 (95% CI, 0.62-0.81) for RFS and 0.72 (95% CI, 0.59-0.85) for OS; the concordance indices in the total neoadjuvant therapy with short-course radiotherapy group were 0.62 (95% CI, 0.49-0.75) for RFS and 0.67 (95% CI, 0.46-0.84) for OS. Calibration plots confirmed good agreement between predicted and observed events. These results compared favorably with predictions based on the AJCC staging system (concordance indices for MSK validation: RFS = 0.69 [95% CI, 0.64-0.74]; OS = 0.67 [95% CI, 0.58-0.75]) and the NAR score (concordance indices for MSK validation: RFS = 0.56 [95% CI, 0.50-0.63]; OS = 0.56 [95% CI, 0.46-0.66]). Furthermore, the clinical calculators provided more individualized outcome estimates compared with the categorical schemas (eg, estimated RFS for patients with AJCC stage IIIB disease ranged from 7% to 68%).

**Conclusions and Relevance:**

In this prognostic study, clinical calculators were developed and validated; these calculators provided more individualized estimates of the likelihood of RFS and OS than the AJCC staging system or the NAR score among patients with rectal cancer who received multimodal treatment. The calculators were easy to use and applicable to both short- and long-course radiotherapy regimens, and they may be used to inform surveillance strategies and facilitate future clinical trials and statistical power calculations.

## Key Points

**Question:**

Do clinical calculators provide more useful estimates of the likelihood of rectal cancer recurrence and patient survival than American Joint Committee on Cancer staging and neoadjuvant rectal score?

**Findings:**

In this prognostic study of 1400 patients with locally advanced rectal cancer, the use of clinical calculators incorporating pathological response, postoperative pathological tumor category, number of positive lymph nodes, tumor location, and the presence of venous and perineural invasion (plus patient age for predicting overall survival) more accurately estimated recurrence-free and overall survival.

**Meaning:**

The results of this study suggest that among patients with rectal cancer who receive multimodal treatment, these clinical calculators may be used to inform posttreatment surveillance strategies and aid risk stratification in clinical trials.

## Teaser

This prognostic study develops and validates clinical calculators to predict recurrence-free survival and overall survival among patients with locally advanced rectal cancer treated with chemotherapy, radiotherapy, and surgery.

## Introduction

The adoption of multimodal therapy for locally advanced (tumor categories 3-4 or node categories 1-2) rectal cancer, including preoperative chemotherapy and radiotherapy, has produced improvements in cancer outcomes.^1,2,3^ Tumor regression facilitates margin-negative resection and is associated with lower rates of local recurrence.^1^ The range of tumor response to neoadjuvant therapy varies,^4^ and patients with complete clinical response (ie, no radiographic or endoscopic evidence of tumor) may be candidates for observation without surgery, often referred to as watch-and-wait or nonoperative management.^5,6^ Most patients have a less-than-complete response, and the extent of tumor downsizing and downstaging is prognostic.^4^

Because pretreatment and postoperative staging often differ owing to tumor downstaging, predicting outcomes in patients with rectal cancer after the receipt of neoadjuvant therapy is challenging. The commonly used tumor, node, and metastasis (TNM) classification system of the American Joint Committee on Cancer (AJCC) and the Union for International Cancer Control is based on 3 anatomic features: depth of tumor invasion into the rectal wall, locoregional lymph node metastases, and distant metastases. Using the TNM system for disease staging among patients with tumor regression after neoadjuvant therapy can produce ambiguities. Posttreatment tumor and node classifications and tumor regression grades can estimate recurrence^4,7^ but overlook many other prognostic features.^8^ In addition, grouping patients according to AJCC classification–based or tumor regression grade–based risk categories assumes homogeneity within groups, whereas outcomes often vary widely among patients within each group.^9,10^

Clinical calculators are built on multivariable models that can incorporate a wide variety of clinical, histological, and pathological variables.^11^ The models are not limited to discrete factors and can incorporate continuous variables with nonlinear associations. Because of their superior predictive accuracy compared with traditional staging systems,^9,10^ clinical calculators have recently been endorsed by the AJCC, and the AJCC Molecular Modelers Working Group has developed guidelines for model creation that emphasize performance measures, implementation clarity, and clinical relevance.^12^

The goal of this prognostic study was to develop clinical calculators for predicting recurrence-free survival (RFS) and overall survival (OS) after the receipt of multimodal therapy among patients with locally advanced rectal cancer. The model was validated in separate internal and external cohorts of patients who received long-course chemoradiotherapy as well as short-course radiotherapy followed by chemotherapy and delayed surgery, a form of total neoadjuvant therapy.

## Methods

### Patients and Treatments

This prognostic study was approved by the institutional review boards of the Memorial Sloan Kettering (MSK) Cancer Center in New York, New York, and the Siteman Cancer Center (SCC) of Washington University in St. Louis, Missouri. A waiver of informed consent was granted based on the study’s use of deidentified data sets. This study followed the Transparent Reporting of a Multivariable Prediction Model for Individual Prognosis or Diagnosis (TRIPOD) reporting guideline for prognostic studies.

Prospectively maintained institutional databases were queried for patients with pretreatment rectal adenocarcinoma within 15 cm of the anal verge that was diagnosed as AJCC stage II or III disease via endorectal ultrasonography or magnetic resonance imaging who received treatment between January 1, 1998, and December 31, 2017. Patients in the MSK cohort received chemoradiotherapy followed by surgery and planned adjuvant chemotherapy between January 1, 1998, and December 31, 2014. Patients in the SCC cohort received either (1) chemoradiotherapy, surgery, and adjuvant chemotherapy (chemoradiotherapy group) or (2) short-course radiotherapy, chemotherapy (with either consolidation leucovorin, fluorouracil, and oxaliplatin [FOLFOX] or consolidation capecitabine and oxaliplatin [CAPOX]), and surgery (total neoadjuvant therapy with short-course radiotherapy group) between January 1, 2009, and December 31, 2017. The surgical procedure for all patients was total mesorectal excision. Patients with metastatic disease and those with cancer that was being managed by a watch-and-wait strategy (which was rare during the study period) were excluded. The regimens for chemoradiotherapy with adjuvant chemotherapy and short-course radiotherapy with consolidation chemotherapy (ie, total neoadjuvant therapy) are available in the eMethods in Supplement 1.^13,14^

### Characteristics and Outcomes

Demographic, clinical, and pathological characteristics as well as follow-up data of 1400 patients were retrieved from institutional databases and manually reviewed via the electronic medical record. Pretreatment characteristics included patient age (patient race and ethnicity were not examined because these characteristics were not reliably reported in the data sets), tumor distance from the anal verge, AJCC clinical tumor (cT) classification, and AJCC clinical node (cN) classification. Time from the end of radiotherapy to surgery was also retrieved, as were the following postresection tumor characteristics: AJCC postoperative pathological tumor (ypT) classification, AJCC postoperative pathological node (ypN) classification, presence of lymphovascular large and small venous invasion (venous invasion), presence of perineural invasion (PNI), number of resected lymph nodes, and number of lymph nodes with metastasis (positive lymph nodes). Downstaging was determined using the 5th edition of the *AJCC Cancer Staging Manual* (*AJCC*-*5*)^15^ rather than the 8th edition (*AJCC-8*) because accurate pretreatment discrimination of N1 vs N2 disease is limited by current staging modalities. Downstaging was calculated by comparing pretreatment cT category, cN category, and *AJCC*-*5* disease stage with ypT category, ypN category, and *AJCC*-*5* disease stage.

Postoperative surveillance was performed in accordance with guidelines from the National Comprehensive Cancer Network.^16^ Recurrence was identified based on radiographic evidence (with or without biopsy), colonoscopy results, and serum carcinoembryonic antigen levels.

The outcome measures were RFS and OS. Recurrence-free survival was defined as the period from the date of surgery to the date of recurrence or death, and patients alive without recurrence were censored at the last follow-up. Overall survival was defined as the period from the date of surgery to the date of death associated with any cause, and patients alive were censored at the last follow-up.

### Development of Clinical Calculators

The MSK cohort was randomly split into a model training group (two-thirds of patients) and an internal validation group (one-third of patients), which were stratified by year of surgery to ensure appropriate temporal representation during the study period. Models were developed using the MSK training data set and validated using the MSK validation data set and the 2 SCC data sets from the chemoradiotherapy group and the total neoadjuvant therapy with short-course radiotherapy group.

Because patients with complete pathological response (ypT0N0) after the receipt of neoadjuvant therapy had a substantially lower likelihood of recurrence and a higher rate of survival compared with other patients,^4^ an a priori decision was made to obtain RFS and OS estimates from Kaplan-Meier curves using data from patients with complete pathological response and to create risk models for predicting RFS and OS using data from patients with incomplete pathological response.

For patients with incomplete pathological response, a univariate proportional hazards regression model was used to evaluate the association of baseline characteristics with RFS and OS. We selected clinically relevant, universally measured variables regardless of whether they were significantly associated with outcomes, and we avoided collinearity. An algorithm was then applied to identify the best-fitting model, defined as the model with the highest χ^2^ value.^17^ To permit nonlinear associations, restricted cubic splines were used for continuous variables in the risk model.^18^ For RFS and OS, the final Cox proportional hazards model included ypT category, number of positive lymph nodes, tumor location (distance from the anal verge of <5 cm vs ≥5 cm), presence of venous invasion, and presence of PNI. The OS model also included patient age. Interaction terms between these covariates were not considered in the final models. The proportional hazards assumption was confirmed using the Schoenfeld test, and the graphical diagnostic results were based on scaled Schoenfeld residuals.^19^ The final clinical calculators predicted RFS and OS using Kaplan-Meier curves for patients with complete pathological response (ypT0N0) and risk models for patients with incomplete pathological response, which were graphically shown as nomograms.

### Statistical Analysis

The discriminatory performance of each clinical calculator for RFS and OS was measured with the concordance index using the MSK validation data set and the 2 SCC data sets. The concordance index was calculated using inverse probability weights for up to 80 months for RFS and up to 60 months for OS.^20^ The concordance index represented the probability that, given 2 randomly selected patients, the patient who had a recurrence first had a higher predicted probability of recurrence. Values were interpreted similarly to the area under the receiver operating characteristic curve, with 0.5 corresponding to random chance and 1.0 corresponding to correct predictions for all patients.^21^ A concordance index was estimated for the entire population (patients with complete and incomplete pathological response) for the 2 outcomes, and bootstrap 95% CIs were calculated.^20^

The clinical calculators were also evaluated using calibration curves, which were created by plotting the predicted RFS and OS at 5 years against the observed outcomes for the MSK validation group and the 2 SCC groups. If the points were on or near the 45-degree line, the model was considered to have good calibration, with a predicted outcome that matched the observed outcome. If the points were higher than the 45-degree line, the model was considered to underestimate outcome probabilities. If the points were lower than the 45-degree line, the model was considered to overestimate outcome probabilities.

Using the concordance index, the predictive accuracies of the clinical calculators were compared with the predictive accuracies of the *AJCC-8* staging system^22^ and the neoadjuvant rectal (NAR) score.^23,24^ Although the NAR score was developed as a surrogate end point for clinical trials of total neoadjuvant therapy, the score has been more recently used as a single prognostic factor.^23^ The NAR score was calculated using the AJCC cT classification, the AJCC ypT classification, and the AJCC ypN classification according to the following steps: (1) the ypT category was subtracted from the cT category and then multiplied by 3; (2) the resulting value was subtracted from the product of 5 multiplied by the ypN category; (3) the resulting value was added to 12 and then squared; and (4) the resulting value was divided by 9.61. With 4 possible values for cT (cT1-cT4), 5 possible values for ypT (ypT0-ypT4), and 3 possible values for ypN (ypN0-ypN2), the NAR score could have 24 discrete values between 0 and 100. Because the NAR score was intended to be used as a surrogate end point for clinical trials of rectal cancer, most studies have categorized risk based on 3 NAR score categories (<8 points, 8-16 points, and >16 points) for estimation of outcomes.^24,25,26^

All statistical analyses were performed using SAS software, version 9.4 (SAS Institute Inc), or R software, version 3.6.0 (R Foundation for Statistical Computing). All *P* values were 2-sided, and *P* < .05 was considered statistically significant. Data were analyzed from March 1, 2020, to January 10, 2021.

## Results

### Cohorts

Of 1400 total patients with locally advanced rectal cancer included in the study, 1069 patients received treatment at MSK, and 331 patients received treatment at SCC. Among those in the MSK cohort, 710 patients were assigned to the model training group, and 359 were assigned to the internal validation group. In the SCC cohort, 200 patients were assigned to the chemoradiotherapy group, which received treatment similar to that received by the MSK cohort (chemoradiotherapy, surgery, and adjuvant chemotherapy), and 131 patients were assigned to the total neoadjuvant therapy with short-course radiotherapy group, which received short-course radiotherapy; consolidation leucovorin, fluorouracil, and oxaliplatin (FOLFOX) chemotherapy or consolidation capecitabine and oxaliplatin (CAPOX) chemotherapy; and surgery.

Among all patients, the median age was 57.8 years (range, 18.0-91.9 years); 863 patients (61.6%) were male, and 537 patients (38.4%) were female, with tumors at a median distance of 6.7 cm (range, 0-15.0 cm) from the anal verge (Table). Preoperative clinical stages (AJCC cT and cN categories) did not differ significantly between the MSK and SCC cohorts, with cT3N1/2 disease being the most common (eg, cT3: 865 patients [80.9%] in the MSK cohort vs 261 patients [78.9%] in the SCC cohort; cN1/2: 724 patients [67.7%] in the MSK cohort vs 236 patients [71.3%] in the SCC cohort). The median time from the end of radiotherapy to surgery was 7.1 weeks (range, 2.0-9.7 weeks) in the MSK training group, 7.1 weeks (range, 4.0-19.9 weeks) in the MSK validation group, 9.0 weeks (range, 2.9-19.9 weeks) in the SCC chemoradiotherapy group, and 18.7 weeks (range, 12.4-32.0 weeks) in the SCC total neoadjuvant therapy with short-course radiotherapy group. Patients were also classified based on complete pathological response (212 patients [19.8%] in the MSK cohort vs 65 patients [19.6%] in the SCC cohort with complete pathological response), AJCC ypT category (eg, 308 patients [28.8%] in the MSK cohort vs 92 patients [27.8%] in the SCC cohort with ypT2 disease), AJCC ypN category (eg, 805 patients [75.3%] in the MSK cohort vs 235 patients [71.0%] in the SCC cohort with ypN0 disease), *AJCC-8* pathological stage (eg, 303 patients [28.3%] in the MSK cohort vs 90 patients [27.2%] in the SCC cohort with stage I disease), number of lymph nodes evaluated (median, 13 nodes [range, 0-47 nodes] in the MSK training set, 14 nodes [range, 1-107 nodes] in the MSK validation set, 14 nodes [range, 0-42 nodes] in the SCC chemoradiotherapy group, and 15 nodes [range, 0-41 nodes] in the SCC short-course radiotherapy with consolidation chemotherapy group), number of positive lymph nodes (median, 2 nodes [range, 1-18 nodes] in the MSK training set, 2 nodes [range, 1-22 nodes] in the MSK validation set, 2 nodes [range, 1-9 nodes] in the SCC chemoradiotherapy group, and 2 nodes [range, 1-8 nodes] in the SCC short-course radiotherapy with consolidation chemotherapy group), presence of venous invasion (139 patients [13.0%] in the MSK cohort vs 42 patients [12.7%] in the SCC cohort with venous invasion), presence of PNI (163 patients [15.2%] in the MSK cohort vs 42 patients [12.7%] in the SCC cohort with PNI), and downstaging (eg, 655 patients [61.3%] in the MSK cohort vs 210 patients [63.4%] in the SCC cohort with *AJCC-5* downstaging).

**Table.** Patient and Disease Characteristics

Characteristic	Patients, No. (%)			
	MSK cohort		SCC cohort	
	Model training group	Validation group	Chemoradiotherapy validation group	Short-course radiotherapy validation group
Total patients, No.	710	359	200	131
Age, median (range), y	58.0 (18.0-89.0)	58.0 (24.0-87.0)	57.3 (31.3-91.9)	57.3 (33.2-85.0)
Sex				
Female	276 (38.9)	148 (41.2)	70 (35.0)	43 (32.8)
Male	434 (61.1)	211 (58.8)	130 (65.0)	88 (67.2)
DTAV, median (range), cm	7 (0-15)	6 (1-15)	6 (0-14)	8 (0-14)
Time from radiotherapy to surgery, median (range), wk	7.1 (2.0-9.7)	7.1 (4.0-19.9)	9.0 (2.9-19.9)	18.7 (12.4-32.0)
cT category				
1	5 (0.7)	1 (0.3)	1 (0.5)	1 (0.8)
2	39 (5.5)	23 (6.4)	16 (8.0)	10 (7.6)
3	580 (81.7)	285 (79.4)	156 (78.0)	105 (80.2)
4	19 (2.7)	16 (4.5)	24 (12.0)	14 (10.7)
Unknown	67 (9.4)	34 (9.5)	3 (1.5)	1 (0.8)
cN category				
0	148 (20.8)	82 (22.8)	63 (31.5)	31 (23.7)
1/2	485 (68.3)	239 (66.6)	136 (68.0)	100 (76.3)
Unknown	77 (10.8)	38 (10.6)	1 (0.5)	0
Pretreatment *AJCC-5* clinical stage				
II	148 (20.8)	82 (22.8)	61 (30.5)	30 (22.9)
III	485 (68.3)	239 (66.6)	136 (68.0)	100 (76.3)
Unknown	77 (10.8)	38 (10.6)	3 (1.5)	1 (0.8)
Pathological tumor response				
Complete	141 (19.9)	71 (19.8)	34 (17.0)	31 (23.7)
Incomplete	567 (79.9)	288 (80.2)	166 (83.0)	100 (76.3)
Unknown	2 (0.3)	0	0	0
ypT category				
0	146 (20.6)	72 (20.1)	37 (18.5)	34 (26.0)
1	46 (6.5)	28 (7.8)	12 (6.0)	12 (9.2)
2	207 (29.2)	101 (28.1)	48 (24.0)	44 (33.6)
3	294 (41.4)	145 (40.4)	93 (46.5)	38 (29.0)
4	16 (2.3)	13 (3.6)	10 (5.0)	3 (2.3)
Unknown	1 (0.1)	0	0	0
ypN category				
0	534 (75.2)	271 (75.5)	139 (69.5)	96 (73.3)
1	133 (18.7)	64 (17.8)	43 (21.5)	30 (22.9)
2	41 (5.8)	24 (6.7)	18 (9.0)	5 (3.8)
Unknown	2 (0.3)	0	0	0
Pathological *AJCC-8* stage				
0	141 (19.9)	71 (19.8)	34 (17.0)	31 (23.7)
I	196 (27.6)	107 (29.8)	49 (24.5)	41 (31.3)
IIA	186 (26.2)	85 (23.7)	52 (26.0)	22 (16.8)
IIB	11 (1.5)	8 (2.2)	4 (2.0)	2 (1.5)
IIIA	54 (7.6)	18 (5.0)	11 (5.5)	16 (12.2)
IIIB	111 (15.6)	64 (17.8)	42 (21.0)	18 (13.7)
IIIC	9 (1.3)	6 (1.7)	8 (4.0)	1 (0.8)
Unknown	2 (0.3)	0	0	0
Lymph nodes, median (range), No.				
Evaluated	13 (0-47)	14 (1-107)	14 (0-42)	15 (0-41)
Positive^a^	2 (1-18)	2 (1-22)	2 (1-9)	2 (1-8)
Venous invasion^b^				
Absent	612 (86.2)	306 (85.2)	163 (81.5)	113 (86.3)
Present	88 (12.4)	51 (14.2)	27 (13.5)	15 (11.5)
Unknown	10 (1.4)	2 (0.6)	10 (5.0)	3 (2.3)
PNI				
Absent	592 (83.4)	293 (81.6)	152 (76.0)	112 (85.5)
Present	104 (14.6)	59 (16.4)	29 (14.5)	13 (9.9)
Unknown	14 (2.0)	7 (1.9)	19 (9.5)	6 (4.6)
Change in tumor category^c^				
Downstaged	358 (50.4)	178 (49.6)	96 (48.0)	90 (68.7)
No change	272 (38.3)	141 (39.3)	96 (48.0)	36 (27.5)
Upstaged	13 (1.8)	6 (1.7)	5 (2.5)	4 (3.1)
Missing data	67 (9.4)	34 (9.5)	3 (1.5)	1 (0.8)
Change in node category^c^				
Downstaged	361 (50.8)	185 (51.5)	95 (47.5)	83 (63.4)
No change	219 (30.8)	103 (28.7)	84 (42.0)	40 (30.5)
Upstaged	52 (7.3)	33 (9.2)	20 (10.0)	8 (6.1)
Missing data	78 (11.0)	38 (10.6)	1 (0.5)	0
Change in *AJCC-5* stage^c^^,^^d^				
Downstaged	435 (61.3)	220 (61.3)	122 (61.0)	88 (67.2)
No change	177 (24.9)	84 (23.4)	63 (31.5)	37 (28.2)
Upstaged	30 (4.2)	21 (5.8)	15 (7.5)	6 (4.6)
Missing data	68 (1.0)	34 (9.5)	0	0

Abbreviations: *AJCC-5*, *AJCC Cancer Staging Manual*, 5th edition^15^; *AJCC-8*, *AJCC Cancer Staging Manual*, 8th edition^22^; cN, clinical node; cT, clinical tumor; DTAV, distance from anal verge; MSK, Memorial Sloan Kettering Cancer Center; PNI, perineural invasion; SCC, Siteman Cancer Center; ypN, postoperative pathological nodal category; ypT, postoperative pathological tumor category.

^a^ Among patients with positive lymph nodes.

^b^ Venous invasion represents small lymphatic and venous invasion, large intramural venous invasion, and large extramural venous invasion.

^c^ Change after neoadjuvant therapy. Upstaging indicates that postoperative pathological category or stage was higher than pretreatment clinical category or stage; downstaging indicates that postoperative pathological category or stage was lower than pretreatment clinical category or stage.

^d^ Complete pathological response was considered downstaging when the initial cancer stage was missing.

### Outcomes and Clinical Calculator Variables

**Figure 1.**
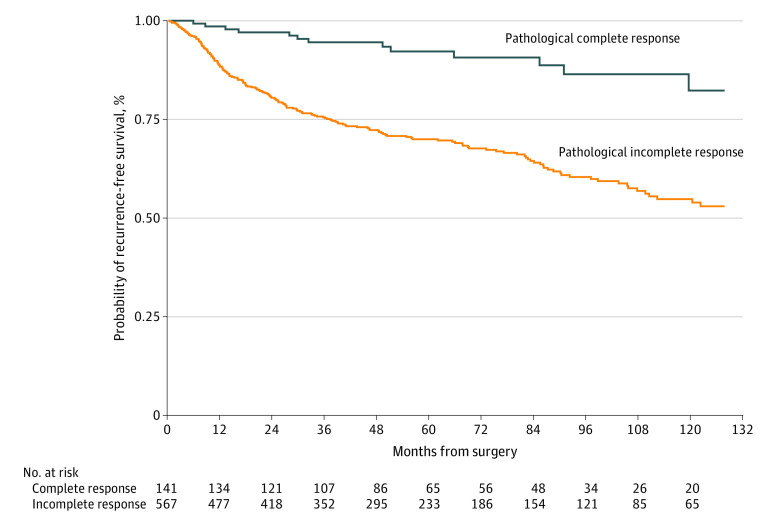
Kaplan-Meier 5-Year Recurrence-Free Survival Among Patients With Complete vs Incomplete Pathological Response to Adjuvant Chemoradiotherapy Survival among patients in the Memorial Sloan Kettering Cancer Center cohort (training data set).

**Figure 2.**
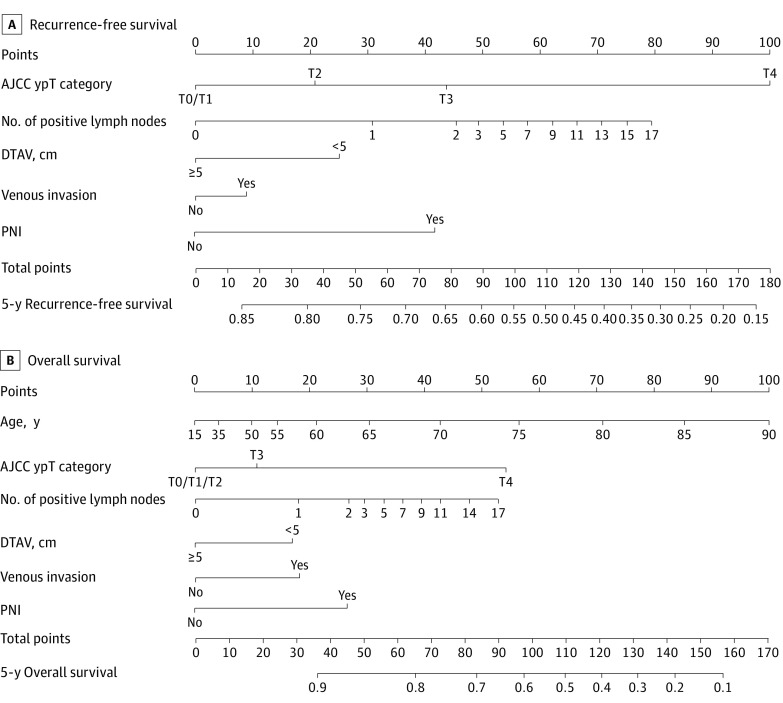
Nomogram of Risk Model for Predicting Recurrence-Free Survival and Overall Survival Survival among patients with incomplete pathological response to neoadjuvant chemoradiotherapy in the Memorial Sloan Kettering Cancer Center cohort (training data set). The nomogram can be interpreted as follows: (1) for each prognostic variable, draw a straight line up to the points axis to determine the points for that variable, (2) repeat this process for each variable, (3) add the points for all variables and locate the sum on the total points axis, and (4) draw a straight line from total points down to 5-year recurrence-free survival or 5-year overall survival. Venous invasion includes small lymphatic and venous invasion, large intramural venous invasion, and large extramural venous invasion. AJCC indicates American Joint Committee on Cancer; DTAV, distance from the anal verge; PNI, perineural invasion; and ypT, postoperative pathological tumor.

Of 141 patients in the MSK training group with complete pathological response (ie, ypT0N0), 17 patients (12.1%) had a recurrence, and 15 patients (10.6%) died. Based on Kaplan-Meier estimates, the 5-year RFS and OS among 833 patients with complete pathological response were 92.0% and 93.0%, respectively (Figure 1 and Figure 2). Of the remaining 567 patients with incomplete pathological response, 198 patients (34.9%) had a recurrence, and 148 patients (26.1%) died, corresponding to a 5-year RFS and OS of 70.0% and 82.0%, respectively.

Independent factors associated with RFS that were included in the risk models for patients with incomplete pathological response were ypT classification, number of positive lymph nodes, distance from the anal verge, presence vs absence of venous invasion, and presence vs absence of PNI (eTable in Supplement 1). The ypT1 category was grouped with the ypT0 category to create a more parsimonious RFS model (Figure 2A).

Independent factors associated with OS that were included in the risk models for patients with incomplete pathological response were age, ypT classification, number of positive lymph nodes, distance from the anal verge, presence vs absence of venous invasion, and presence vs absence of PNI (eTable in Supplement 1). The ypT0, ypT1, and ypT2 categories were grouped together to create a more parsimonious OS model (Figure 2B). Equations used to predict RFS and OS among those with incomplete pathological response are provided in the eFigure in Supplement 1.

### Validation

The RFS and OS clinical calculators for patients with complete vs incomplete pathological response were validated using data from the MSK validation group and the 2 SCC validation groups (ie, the chemoradiotherapy group and the total neoadjuvant therapy with short-course radiotherapy group). The discriminatory performance of the clinical calculators, the *AJCC-8* staging system, and the NAR score were measured with the concordance index.

For predicting RFS in the MSK validation group, the concordance indices were 0.70 (95% CI, 0.65-0.76) for the clinical calculator, 0.69 (95% CI, 0.64-0.74) for the *AJCC-8* staging system, and 0.56 (95% CI, 0.50-0.63) for the NAR score. For predicting RFS in the SCC chemoradiotherapy group, the concordance index was 0.71 (95% CI, 0.62-0.81) for the clinical calculator, 0.68 (95% CI, 0.60-0.75) for the *AJCC-8* staging system, and 0.67 (95% CI, 0.59-0.76) for the NAR score. For predicting RFS in the SCC total neoadjuvant therapy with short-course radiotherapy group, the concordance indices were 0.62 (95% CI, 0.49-0.75) for the clinical calculator, 0.63 (95% CI, 0.52-0.73) for the *AJCC-8* staging system, and 0.60 (95% CI, 0.49-0.73) for the NAR score.

For predicting OS in the MSK validation group, the concordance indices were 0.73 (95% CI, 0.65-0.80) for the clinical calculator, 0.67 (95% CI, 0.58-0.75) for the *AJCC-8* staging system, and 0.56 (95% CI, 0.46-0.66) for the NAR score. For predicting OS in the SCC chemoradiotherapy group, the concordance indices were 0.72 (95% CI, 0.59-0.85) for the clinical calculator, 0.64 (95% CI, 0.53-0.74) for the *AJCC-8* staging system, and 0.69 (95% CI, 0.57-0.81) for the NAR score. For predicting OS in the SCC total neoadjuvant therapy with short-course radiotherapy group, the concordance indices were 0.67 (95% CI, 0.46-0.84) for the clinical calculator, 0.60 (95% CI, 0.45-0.77) for the *AJCC-8* staging system, and 0.60 (95% CI, 0.47-0.79) for the NAR score.

**Figure 3.**
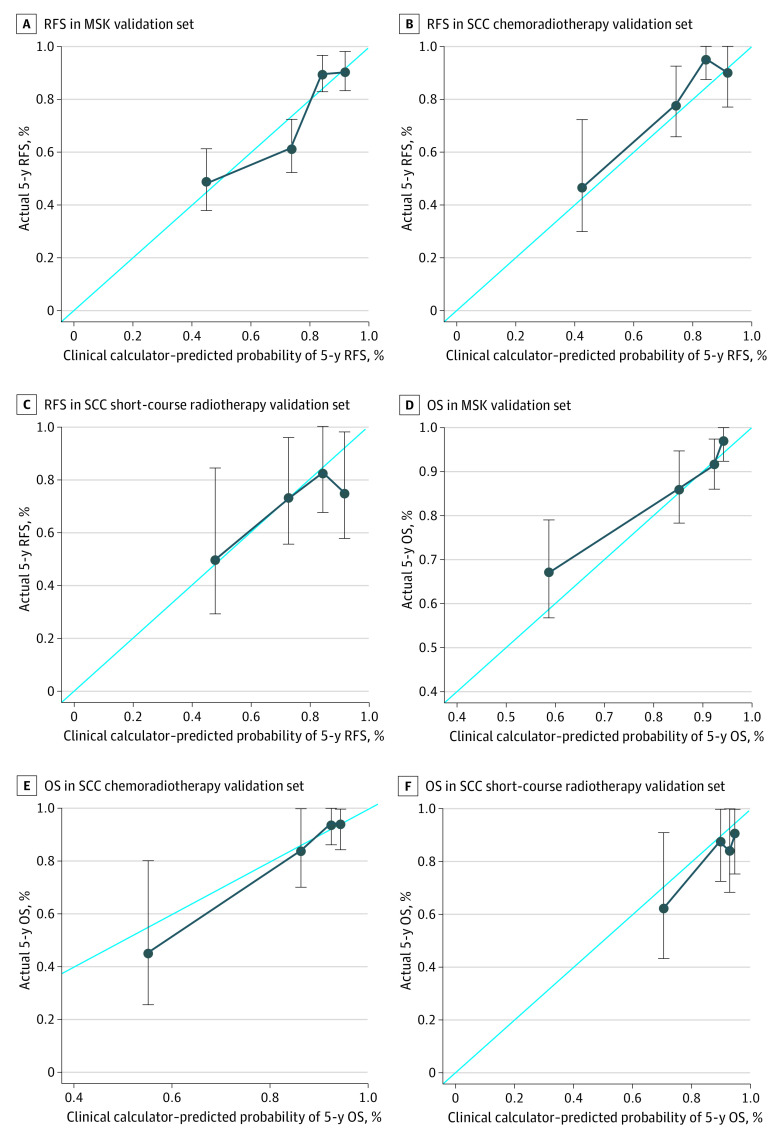
Calibration Curves for 5-Year Recurrence-Free Survival (RFS) and Overall Survival (OS) One Siteman Cancer Center (SCC) group received chemoradiotherapy, and the other group received short-course radiotherapy with consolidation chemotherapy and surgery. Error bars represent 95% CIs of the observed 5-year survival rate determined by the Kaplan-Meier method. MSK indicates Memorial Sloan Kettering Cancer Center.

**Figure 4.**
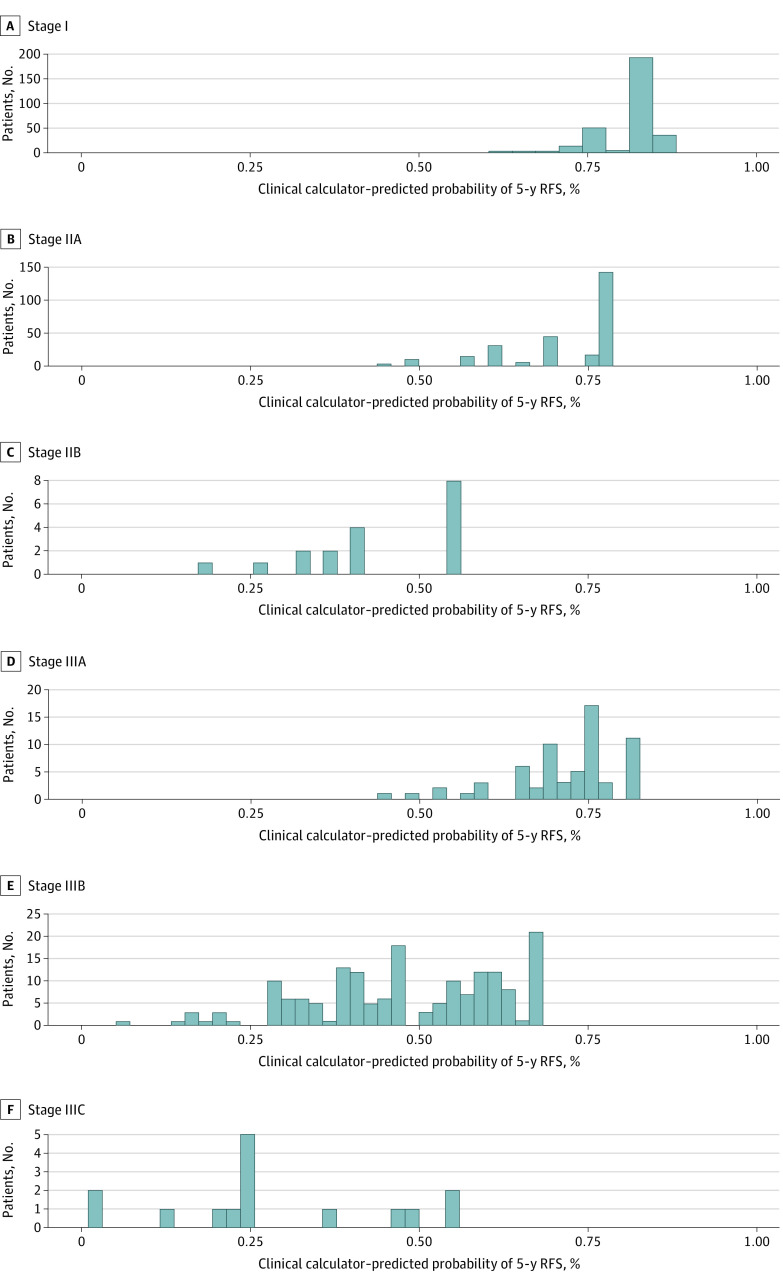
Heterogeneity of Clinical Calculator–Predicted Probabilities of 5-Year Recurrence-Free Survival (RFS) in the Memorial Sloan Kettering Cancer Center Cohort Survival within each disease substage. Substages were categorized based on the *AJCC Cancer Staging Manual*, 8th edition.^22^ Of 1069 total patients, 291 had stage I disease, 265 had stage IIA disease, 18 had stage IIB disease, 65 had stage IIIA disease, 171 had stage IIIB disease, and 15 had stage IIIC disease.

The calibration plots for predicting 5-year RFS and OS using the clinical calculators in the validation data sets are shown in Figure 3. The plot of predicted outcome (RFS or OS) vs observed outcome (RFS or OS) approximated a 45-degree diagonal for all 3 validation data sets. The range of 5-year RFS estimates within each *AJCC-8* disease substage for the 1069 patients in the MSK cohort is shown in Figure 4. For example, the RFS for patients with AJCC stage IIIB cancer ranged from 7% to 68%.

## Discussion

In this prognostic study, the MSK clinical calculators, which incorporated clinical, histological, and AJCC pathological variables, outperformed the *AJCC-8* staging system and the NAR score for predicting RFS and OS among patients with locally advanced rectal cancer who received multimodal treatment. The concordance indices of the clinical calculators were modestly higher than those of the *AJCC-8* staging system; however, the *AJCC-8* categorical system assumes homogeneity and cannot discriminate between outcomes within disease substages. In contrast, the MSK clinical calculators could discriminate errant outcomes within each substage by providing continuous estimates of risk. For example, among patients in the MSK cohort who had AJCC stage IIIB disease (67% of patients with stage III disease), the 5-year RFS was 48%, but the clinical calculator–predicted RFS for patients with stage IIIB disease ranged from 7% to 68% (Figure 4). Thus, the MSK clinical calculators provided more individualized data via judicious use of all available relevant information.

The outperformance of the NAR score by our clinical calculators was consistent with the findings of a recent study outlining the limitations of the NAR score.^25^ In addition to the restrictions imposed by a categorical system, the inadequate performance of the clinical calculators may be associated with inaccurate pretreatment clinical staging.^1^ Response to neoadjuvant therapy is a measure of tumor biology that is not fully captured by baseline characteristics and is more prognostic than pretreatment clinical staging.^4^

Radiotherapy has been used for the treatment of rectal cancer for the past 4 decades with the aim of reducing high rates of local recurrence after rectal resection. Prospective randomized clinical trials have demonstrated that postoperative chemoradiotherapy (45-54 Gy delivered in 25-28 fractions with radiosensitizing fluorouracil) can significantly reduce the likelihood of local recurrence after rectal cancer resection.^2^ Preoperative chemoradiotherapy was subsequently reported to further reduce the likelihood of local recurrence and lessen toxic effects.^1^ In those studies,^1,2^ surgery was routinely performed 6 weeks after the receipt of neoadjuvant therapy, and rectal cancer response was evident in tumor downsizing, tumor downstaging, and tumor regression.^7^

In short-course radiotherapy, a total dose of 25 Gy is administered in 5 fractions, which is biologically equivalent to long-course radiotherapy. Early studies found that performing surgery immediately after receipt of short-course radiotherapy did not allow sufficient time for tumor downsizing or downstaging but reduced the likelihood of local recurrence.^3,27^ A later study reported that delaying surgery after short-course radiotherapy allowed the tumor to respond,^28^ and the addition of consolidation chemotherapy, which was implemented in the total neoadjuvant therapy arm of the RAPIDO (Rectal Cancer and Preoperative Induction Therapy Followed by Dedicated Operation) clinical trial, further enhanced the response.^29^

Although our clinical calculators were developed using data from patients who received neoadjuvant chemoradiotherapy, the calculators are likely broadly applicable because they were validated using data from patients who received short-course radiotherapy with consolidation chemotherapy, a form of total neoadjuvant therapy.^29^ Only a few previous clinical calculators developed to predict rectal cancer recurrence and patient survival have undergone appropriate external validation. One such calculator, developed by Valentini et al^30^ for predicting rectal cancer recurrence, metastasis, and patient survival after radiotherapy or chemoradiotherapy (based on data from European phase III clinical trials conducted between 1992 and 2003), includes dose of radiotherapy, concomitant use of chemotherapy and radiotherapy, adjuvant chemotherapy, clinical and pathological AJCC tumor and node categories, and procedure performed (rather than specific location of the tumor). The concordance index for that clinical calculator was similar to the indices of our clinical calculators, suggesting comparable discrimination; however, the Valentini et al^30^ study had statistical limitations because it performed imputation without penalty and did not address the competing risk of local and distant recurrence. Furthermore, the model in that study was less applicable to patients receiving contemporary standard radiotherapy and chemotherapy regimens as well as newer multimodal treatments, such as total neoadjuvant therapy.

The lower predictive accuracy (as measured by the concordance index) of the clinical calculators compared with those of colon cancer models^31,32^ may be associated with tumor response to multimodality therapy and represents an opportunity for improvement, possibly with the future addition of molecular and/or radiographic variables to the model, which capture the dynamics of tumor regression. The present study designed a web interface^33^ incorporating estimates from Kaplan-Meier curves for patients with complete response and risk models (depicted using nomograms) for patients with incomplete response to provide an easy-to-use method for patients and physicians to calculate the likelihood of 5-year RFS and OS.

### Limitations

This study has limitations. It is subject to the selection bias inherent in any retrospective study, which we minimized by closely following the methodological criteria established by the AJCC Precision Medicine Core.^12^ This adherence to standards is especially important given that most prognostic tools for colorectal cancer have methodological deficiencies.^34^ Other strengths include the relatively large cohort of patients who underwent standardized resection procedures, comprehensive histological assessment by specialized pathologists, and the availability of granular clinical and demographic information. Another advantage of the study is the rigorous validation performed using both internal (MSK) and external (SCC) independent data sets, which addresses the common concern about the applicability of risk models developed from single-institution data. The validation results suggest that our predictions may be relevant to the general population.

## Conclusions

In this prognostic study, the clinical calculators provided more individualized estimates of the likelihood of rectal cancer recurrence and patient survival than the AJCC staging system or the NAR score. These estimates can be used to inform surveillance routines and aid risk stratification in clinical trials.

## Notes

Supplement 1. **eMethods.** Treatment Regimens **eTable.** Parameter Estimates for Cox Regression Models Predicting RFS and OS **eFigure.** Predictive Equations for Incomplete Responders for RFS and OS

Supplement 2. **Nonauthor Collaborators.** Members of the Colorectal Cancer Disease Management Team of Memorial Sloan Kettering Cancer Center
