# Errors in a Supplement

**DOI:** 10.1001/jamanetworkopen.2021.47251

**Published:** 2022-01-13

In the Original Investigation “Development and Assessment of a Clinical Calculator for Estimating the Likelihood of Recurrence and Survival Among Patients With Locally Advanced Rectal Cancer Treated With Chemotherapy, Radiotherapy, and Surgery,”^1^ published November 8, 2021, a supplement has been amended to fix the spelling of 3 nonauthor collaborator names. This article has been corrected.^1^
