# Supplementary material for: Development and Assessment of a Clinical Calculator for Estimating the Likelihood of Recurrence and Survival Among Patients With Locally Advanced Rectal Cancer Treated With Chemotherapy, Radiotherapy, and Surgery
Source: JAMA Netw Open. 2021 Nov 8;4(11):e2133457. doi: 10.1001/jamanetworkopen.2021.33457 (PMC8576585; doi:10.1001/jamanetworkopen.2021.33457)
Supplement: Supplement 1. — eMethods. Treatment Regimens eTable. Parameter Estimates for Cox Regression Models Predicting RFS and OS eFigure. Predictive Equations for Incomplete Responders for RFS and OS [file jamanetwopen-e2133457-s001.pdf]

Supplementary Online Content

Weiser MR, Chou JF, Keshinro A, et al; Colorectal Cancer Disease Management Team of Memorial Sloan Kettering Cancer Center. Development and assessment of a clinical calculator for estimating the likelihood of recurrence and survival among patients with locally advanced rectal cancer treated with chemotherapy, radiotherapy, and surgery. *JAMA Netw Open*. 2021;4(11):e2133457. doi:10.1001/jamanetworkopen.2021.33457

eMethods. Treatment Regimens

eTable. Parameter Estimates for Cox Regression Models Predicting RFS and OS

eFigure. Predictive Equations for Incomplete Responders for RFS and OS

This supplementary material has been provided by the authors to give readers additional information about their work.

© 2021 Weiser MR et al. *JAMA Network Open*.

eMethods. Treatment Regimens

Treatment Regimens

Chemoradiotherapy consisted of 25 to 28 radiotherapy fractions with concurrent infusional fluorouracil at 225 mg/m^2 or oral capecitabine at 825 mg/m^2 twice daily on days of radiation. The radiotherapy was in the form of three-dimensional conformal radiotherapy or intensity-modulated radiotherapy, for a total dose of 45 Gy in 1.8-Gy fractions to the pelvis, with a 5.4-Gy boost to the tumor. In three-dimensional conformal radiotherapy, the boost involved three additional 1.8-Gy fractions (for a total dose of 50.4 Gy in 28 fractions), whereas in intensity-modulated radiotherapy the boost involved dose painting to 2.0 Gy/fraction (for a total dose of 50 Gy in 25 fractions). Adjuvant therapy, generally mFOLFOX for eight cycles or CAPOX for five cycles, was delivered in accordance with the national guidelines (www.nccn.org). Prior to 2004, adjuvant therapy consisted of fluorouracil and leucovorin. Short-course radiotherapy consisted of 25 Gy of three-dimensional conformal radiotherapy delivered over 5 days and was followed by 4 months of modified FOLFOX or CAPOX and then total mesorectal excision.

© 2021 Weiser MR et al. *JAMA Network Open*.

eTable. Parameter Estimates for Cox Regression Models Predicting RFS and OS

RFS

Variable	HR (95%CI)
ypT0/T1	Ref
ypT2 vs ypT0/T1	1.37 (0.67-2.79)
ypT3 vs ypT0/T1	1.94 (0.97-3.86)
ypT4 vs ypT0/T1	4.58 (1.70-12.4)
Positive LN	NDI
DTAV >=5 vs <5	0.68 (0.49-0.94)
LVI Yes vs No	1.15 (0.77-1.69)
PNI Yes vs No	1.88 (1.32-2.70)

OS

Variable	HR (95%CI)
Age	NDI*
ypT3 vs ypT0/T1/T2	1.48 (1.07-2.04)
ypT4 vs ypT0/T1/T2	3.80 (1.72-8.41)
Positive LN	NDI
DTAV >=5 vs <5	0.69 (0.50-0.96)
LVI Yes vs No	1.09 (0.73-1.62)
PNI Yes vs No	2.02 (1.41-2.91)

DTAV, distance of tumor to anal verge; HR, hazard ratio; LN, lymph node; venous invasion, large and small vessel lymphovascular invasion; OS, overall survival; PNI, perineural invasion; RFS, relapse-free survival; T, pathologic T category; ypT, pathologic AJCC tumor classification NDI, no direct interpretation of HRs because the continuous variable was modeled using restricted cubic splines

© 2021 Weiser MR et al. *JAMA Network Open*.

eFigure. Predictive Equations for Incomplete Responders for RFS (top) and OS (bottom)

RFS

$$\text{Prob}\{T \geq t\} = S_0(t)^{e^{X\beta}}, \quad \text{where}$$

$$\begin{aligned}
X\hat{\beta} = \\
& -0.5425329 \\
& +0.3143759[\text{T2}] + 0.6630723[\text{T3}] + 1.521675[\text{T4}] \\
& +0.522283\text{posLN} - 0.05440106(\text{posLN})_+^3 + 0.08160159(\text{posLN} - 1)_+^3 \\
& -0.02720053(\text{posLN} - 3)_+^3 \\
& -0.3811237[\text{DTAV} \;\; >= 5] + 0.136099\ \text{VenousInvasion} + 0.6336823\ \text{PNI}
\end{aligned}$$

and $[c] = 1$ if subject is in group c, 0 otherwise; $(x)_+ = x$ if $x > 0$, 0 otherwise

t	$S_0(t)$
0	1.000
60	0.719
120	0.560
180	0.320

OS

$$\text{Prob}\{T \geq t\} = S_0(t)^{e^{X\beta}}, \quad \text{where}$$

$$\begin{aligned}
X\hat{\beta} = \\
& -0.9603734 \\
& +0.00535203\text{AGERX} + 2.213425\times10^{-5}(\text{AGERX} - 36)_+^3 - 1.059292\times10^{-5}(\text{AGERX} - 52)_+^3 \\
& -4.505452\times10^{-5}(\text{AGERX} - 64)_+^3 + 3.351319\times10^{-5}(\text{AGERX} - 78.7)_+^3 \\
& +0.2726462[\text{T3}] + 1.386825[\text{T4}] \\
& +0.5150229\text{posLN} - 0.05251174(\text{posLN})_+^3 + 0.07876761(\text{posLN} - 1)_+^3 \\
& -0.02625587(\text{posLN} - 3)_+^3 \\
& -0.4471371[\text{DTAV} \;\; >= 5] + 0.470128\ \text{VenousInvasion} + 0.6762158\ \text{PNI}
\end{aligned}$$

and $[c] = 1$ if subject is in group c, 0 otherwise; $(x)_+ = x$ if $x > 0$, 0 otherwise

t	$S_0(t)$
0	1.000
60	0.854
120	0.654
180	0.420

© 2021 Weiser MR et al. *JAMA Network Open*.
