# Supplementary material for: Development and Assessment of a Clinical Calculator for Estimating the Likelihood of Recurrence and Survival Among Patients With Locally Advanced Rectal Cancer Treated With Chemotherapy, Radiotherapy, and Surgery
Source: JAMA Netw Open. 2021 Nov 8;4(11):e2133457. doi: 10.1001/jamanetworkopen.2021.33457 (PMC8576585; doi:10.1001/jamanetworkopen.2021.33457)
Supplement: Supplement 2. — Nonauthor Collaborators. Members of the Colorectal Cancer Disease Management Team of Memorial Sloan Kettering Cancer Center [file jamanetwopen-e2133457-s002.pdf]

Supplemental Online Content: Nonauthor Collaborators

*Indicates required information. Only first name, last name, and suffix will appear in PubMed.

*Group Name(s): Colorectal Cancer Disease Management Team of Memorial Sloan Kettering Cancer Center							
***First Name and Middle Initial(s)**	***Last Name**	***Suffix (eg, Jr, III)**	Academic Degrees	Institution	Location (city, state/province, country)	Role or Contribution, eg, chair, principal investigator	Group (if more than 1 Group listed in the byline) and/or Subgroup (eg, Steering Committee)
Fiyinfolu	Balogun		MD	Memorial Sloan Kettering Cancer Center	New York, NY, USA		
David	Bates		MD	Memorial Sloan Kettering Cancer Center	New York, NY, USA		
Delia	Calo		MD	Memorial Sloan Kettering Cancer Center	New York, NY, USA		
Louise	Connell		MD	Memorial Sloan Kettering Cancer Center	New York, NY, USA		
John	Cuaron		MD	Memorial Sloan Kettering Cancer Center	New York, NY, USA		
Avni	Desai		MD	Memorial Sloan Kettering Cancer Center	New York, NY, USA		
Natally	deSouza Horvat		MD	Memorial Sloan Kettering Cancer Center	New York, NY, USA		
Imane	El Dika		MD	Memorial Sloan Kettering Cancer Center	New York, NY, USA		
Maria	El Homsi		MD	Memorial Sloan Kettering Cancer Center	New York, NY, USA		
David	Felek		MD	Memorial Sloan Kettering Cancer Center	New York, NY, USA		
J. Louis	Fuqua	III	MD	Memorial Sloan Kettering Cancer Center	New York, NY, USA		
Karuna	Ganesh		MD	Memorial Sloan Kettering Cancer Center	New York, NY, USA		
Hans	Gerdes		MD	Memorial Sloan Kettering Cancer Center	New York, NY, USA		
Zoe	Goldberg		MD	Memorial Sloan Kettering Cancer Center	New York, NY, USA		
Jennifer	Golia Pernicka		MD	Memorial Sloan Kettering Cancer Center	New York, NY, USA		
Carla	Hajj		MD	Memorial Sloan Kettering Cancer Center	New York, NY, USA		
Jaclyn	Hechtman		MD	Memorial Sloan Kettering Cancer Center	New York, NY, USA		
Dennis	Hsu		MD	Memorial Sloan Kettering Cancer Center	New York, NY, USA		
Sidra	Javed-Tayyab		MD	Memorial Sloan Kettering Cancer Center	New York, NY, USA		
Nancy	Kemeny		MD	Memorial Sloan Kettering Cancer Center	New York, NY, USA		

Supplemental Online Content: Nonauthor Collaborators

*Indicates required information. Only first name, last name, and suffix will appear in PubMed.

***First Name and Middle Initial(s)**	***Last Name**	***Suffix (eg, Jr, III)**	Academic Degrees	Institution	Location (city, state/province, country)	Role or Contribution, eg, chair, principal investigator	Group (if more than 1 Group listed in the byline) and/or Subgroup (eg, Steering Committee)
Jia	Li		MD	Memorial Sloan Kettering Cancer Center	New York, NY, USA		
Emmy	Ludwig		MD	Memorial Sloan Kettering Cancer Center	New York, NY, USA		
Robin	Mendelsohn		MD	Memorial Sloan Kettering Cancer Center	New York, NY, USA		
Parisa	Momtaz		MD	Memorial Sloan Kettering Cancer Center	New York, NY, USA		
Garrett	Nash		MD	Memorial Sloan Kettering Cancer Center	New York, NY, USA		
Makoto	Nishimura		MD	Memorial Sloan Kettering Cancer Center	New York, NY, USA		
Maliha	Nusrat		MD	Memorial Sloan Kettering Cancer Center	New York, NY, USA		
Emmanouil	Pappou		MD	Memorial Sloan Kettering Cancer Center	New York, NY, USA		
Viktoria	Paroder		MD	Memorial Sloan Kettering Cancer Center	New York, NY, USA		
Philip	Paty		MD	Memorial Sloan Kettering Cancer Center	New York, NY, USA		
Iva	Petkovska		MD	Memorial Sloan Kettering Cancer Center	New York, NY, USA		
Nitya	Raj		MD	Memorial Sloan Kettering Cancer Center	New York, NY, USA		
Diane	Reidy		MD	Memorial Sloan Kettering Cancer Center	New York, NY, USA		
Marsha	Reyngold		MD	Memorial Sloan Kettering Cancer Center	New York, NY, USA		
Michael	Roehrl		MD	Memorial Sloan Kettering Cancer Center	New York, NY, USA		
Juan	Schvartzman		MD	Memorial Sloan Kettering Cancer Center	New York, NY, USA		

Supplemental Online Content: Nonauthor Collaborators

*Indicates required information. Only first name, last name, and suffix will appear in PubMed.

***First Name and Middle Initial(s)**	***Last Name**	***Suffix (eg, Jr, III)**	Academic Degrees	Institution	Location (city, state/province, country)	Role or Contribution, eg, chair, principal investigator	Group (if more than 1 Group listed in the byline) and/or Subgroup (eg, Steering Committee)
Mark	Schattner		MD	Memorial Sloan Kettering Cancer Center	New York, NY, USA		
Neil	Segal		MD	Memorial Sloan Kettering Cancer Center	New York, NY, USA		
Armin	Shahrokni		MD	Memorial Sloan Kettering Cancer Center	New York, NY, USA		
Marina	Shcherba		MD	Memorial Sloan Kettering Cancer Center	New York, NY, USA		
Moshe	Shike		MD	Memorial Sloan Kettering Cancer Center	New York, NY, USA		
J. Joshua	Smith		MD	Memorial Sloan Kettering Cancer Center	New York, NY, USA		
Zsofia	Stadler		MD	Memorial Sloan Kettering Cancer Center	New York, NY, USA		
Ryan	Sugarman		MD	Memorial Sloan Kettering Cancer Center	New York, NY, USA		
Efsevia	Vakiani		MD	Memorial Sloan Kettering Cancer Center	New York, NY, USA		
Iris	Wei		MD	Memorial Sloan Kettering Cancer Center	New York, NY, USA		
Maria	Widmar		MD	Memorial Sloan Kettering Cancer Center	New York, NY, USA		
Elizabeth	Won		MD	Memorial Sloan Kettering Cancer Center	New York, NY, USA		
Abraham	Wu		MD	Memorial Sloan Kettering Cancer Center	New York, NY, USA		
Rona	Yaeger		MD	Memorial Sloan Kettering Cancer Center	New York, NY, USA		
Alice	Zervoudakis		MD	Memorial Sloan Kettering Cancer Center	New York, NY, USA		
Melissa	Zinovoy		MD	Memorial Sloan Kettering Cancer Center	New York, NY, USA		
